# Community-based mental health treatments for survivors of torture and militant attacks in Southern Iraq: a randomized control trial

**DOI:** 10.1186/s12888-015-0622-7

**Published:** 2015-10-14

**Authors:** William M. Weiss, Laura K. Murray, Goran Abdulla Sabir Zangana, Zayan Mahmooth, Debra Kaysen, Shannon Dorsey, Kristen Lindgren, Alden Gross, Sarah McIvor Murray, Judith K. Bass, Paul Bolton

**Affiliations:** Department of International Health, Johns Hopkins Bloomberg School of Public Health, 615 N. Wolfe Street, Baltimore, MD 21205 USA; Department of Mental Health, Johns Hopkins Bloomberg School of Public Health, 624 N. Broadway Street, Baltimore, MD 21205 USA; Heartland Alliance International, Rizgary Taza 408, Alley 32, House 08, Sulaimaniay, Iraq; Department of Psychology, University of Washington, 335 Guthrie Hall, Seattle, WA 98195 USA; Center for the Study of Health and Risk Behaviors, University of Washington, 1100 NE 45th, Suite 300, Seattle, WA 98105 USA; Department of Epidemiology, Johns Hopkins Bloomberg School of Public Health, 615 N. Wolfe Street, Baltimore, MD 21205 USA

**Keywords:** Transdiagnostic, Cognitive therapy, Psychological trauma, Global mental health, Randomized controlled trial, Mental health services, Iraq, Torture survivors, Community health workers, Task shifting

## Abstract

**Background:**

Systematic violence is a long-standing problem in Iraq. Research indicates that survivors often experience multiple mental health problems, and that there is a need for more rigorous research that targets symptoms beyond post-traumatic stress (PTS). Our objective was to test the effectiveness of two counseling therapies in Southern Iraq in addressing multiple mental health problems among survivors of systematic violence: (1) a transdiagnostic intervention (Common Elements Treatment Approach or CETA); and (2) cognitive processing therapy (CPT). The therapies were provided by non-specialized health workers since few MH professionals are available to provide therapy in Iraq.

**Methods:**

This was a randomized, parallel, two site, two-arm (1:1 allocation), single-blinded, wait-list controlled (WLC) trial of CETA in one site (99 CETA, 50 WLC), and CPT in a second site (129 CPT, 64 WLC). Eligibility criteria were elevated trauma symptoms and experience of systematic violence. The primary and secondary outcomes were trauma symptoms and dysfunction, respectively, with additional assessment of depression and anxiety symptoms. Non-specialized health workers (community mental health worker, CMHW) provided the interventions in government-run primary health centers. Treatment effects were determined using longitudinal, multilevel models with CMHW and client as random effects, and a time by group interaction with robust variance estimation, to test for the net difference in mean score for each outcome between the baseline and follow up interview. Multiple imputation techniques were used to account for missingness at the item level and the participant level. All analyses were conducted using Stata 12.

**Results:**

The CETA intervention showed large effect sizes for all outcomes. The CPT intervention showed moderate effects sizes for trauma and depression, with small to no effect for anxiety or dysfunction, respectively.

**Conclusions:**

Both CETA and CPT appear to benefit survivors of systematic violence in Southern Iraq by reducing multiple mental health symptoms, with CETA providing a very large benefit across a range of symptoms. Non-specialized health workers were able to treat comorbid symptoms of trauma, depression and anxiety, and dysfunction among survivors of systematic violence who have limited access to mental health professionals. The trial further supports the use of evidence-based therapies in lower-resource settings.

**Trial registration and protocol:**

This trial was registered at ClinicalTrials.gov on 16 July 2010 with an identifier of NCT01177072 as the Study of Effectiveness of Mental Health Interventions among Torture Survivors in Southern Iraq.

The study protocol can be downloaded from the following website: http://tinyurl.com/CETA-Iraq-Protocol. In the protocol, the CETA intervention is given a different name: components-based intervention or CBI.

**Electronic supplementary material:**

The online version of this article (doi:10.1186/s12888-015-0622-7) contains supplementary material, which is available to authorized users.

## Background

Systematic violence, including torture, has been a long-standing problem in Iraq, particularly during the Saddam Hussein era [1–5]. Survivors experience increased risk for multiple adverse mental health outcomes. For example, in a meta-analysis of mental health problems among populations who were displaced or affected by conflict, Steel et al. [6] found that those who reported torture had twice the odds of posttraumatic stress disorder (PTSD) and one and half times the odds of depression. Generalized anxiety is also common among torture survivors [7–10].

Among the mental health therapies for adult survivors of systematic violence that have been evaluated and included in review articles, most assessed PTS as the primary outcome [11–15]. Cognitive behavioral therapy (CBT), narrative exposure therapy (NET), testimony therapy (TT), and/or exposure therapy have been the most frequently studied interventions. One review of treatment for refugees and asylum-seekers found support for the effectiveness of CBT and NET [11]. Another found support only for trauma-focused treatments [12], and a third concluded that exposure-based and CBT-based treatments both showed effectiveness [13]. Prominent across all these reviews were cautions in interpretation due to methodological limitations in the studies reviewed, such as non-random allocation to treatment, lack of controls, and small sample sizes. Given the comorbidity documented among survivors of systematic violence, a recent review of interventions expanded its search beyond participants with just PTS to include a broader population of adults with histories of trauma and/or torture [15]. Of the multiple types of research designs reviewed by McFarlane et al., the authors reported only 11 randomized control trials (RCTs) which examined individual psychotherapies specifically targeting PTS symptoms (NET, CBT, TT and exposure therapy) or healing workshops (1 study) among resettled refugees (5 studies), asylum seekers (1 study), displaced persons (2 studies), and survivors residing within their country of origin (3 studies) [15]. Overall, the RCT-evaluated therapies were effective in reducing PTS symptoms but less consistent in reducing depression or other trauma-related symptoms. This review also highlighted a need to address symptoms beyond PTS and for more rigorous research studies of treatments for torture survivors.

Most evidence-based therapies (EBT) in mental health focus on one disorder (e.g., PTS), although the treatment may have broader effects. This focus on a single mental health disorder presents challenges for implementation and sustainability in low and middle income countries (LMIC) including: (1) limited available resources (e.g., finances, personnel) for training and scaling up multiple EBTs to reduce the treatment gap across multiple disorders; (2) challenges inherent in learning multiple EBTs and implementing them with fidelity; and, (3) the lack of direction for non-specialized health workers on how to deal with comorbidity [16]. Common elements or “transdiagnostic” mental health approaches teach a set of common practice elements that can be delivered in varying combinations to address a range of mental health problems [17, 18]. Decision rules, based on research evidence, guide selection, sequencing, and dosing of elements and allow for flexibility in individual symptom presentation [19, 20]. We developed a transdiagnostic treatment, the Common Elements Treatment Approach [16] for one trial intervention based on the existence of comorbidities in the study population, growing evidence of effectiveness in high-income settings, evidence of greater acceptability in high income settings (e.g., provider attitudes, low drop-out rates), and the limited mental health resources available in Iraq [19, 21–24]. For the other intervention, we chose an established, evidence-based cognitive behavior therapy approach, cognitive processing therapy (CPT), based on preliminary findings of a similar trial in Northern Iraq by several authors [25].

### Objectives

The objective of the current trial was to test the effectiveness of a transdiagnostic intervention, the Common Elements Treatment Approach (CETA) [16] and cognitive processing therapy (CPT), for addressing mental health problems among survivors of systematic violence as provided by non-specialized health workers at the primary health care level. Our hypothesis was that participants receiving CETA or CPT would show significantly more improvement in symptoms of trauma, depression and anxiety, as well as dysfunction, compared to those in the waitlist control (WLC) condition.

## Methods

### Ethical statement

Institutional review boards at the Johns Hopkins Bloomberg School of Public Health, and the Ministry of Health in Iraq’s Psychiatric Research Ethics Committee approved the protocol. Study participants provided oral informed consent, and none received compensation.

### Trial design

This was a parallel, two-site, two-arm (1:1 allocation), single-blinded, wait-list randomized controlled trial. It was single-blinded: interviewers at baseline and follow-up did not know to which study arm the interviewees belonged. This RCT compared a transdiagnostic counseling intervention (CETA) in one site, and CPT in a second site, with separate WLCs in both sites. CETA was provided by 12 non-specialized health workers (eight males, four females), called community mental health workers (CMHWs), working in and around the cities of Karbala, Najaf and Hilla, south of Baghdad. CPT was provided by 17 CMHWs working in and around the cities of Basra and Nassariyah in the far south of Iraq. CMHWs are non-mental health professionals who are trained to provide mental health services locally (i.e., the mental health equivalent of community health workers or CHWs). In this trial, the CMHWs were medics or nurses who worked in rural Ministry of Health primary health care centers. The CMHWs had received training in non-specific counseling methods some years before by our partner international non-governmental organization (Heartland Alliance International) and continued to provide these services part-time.

### Changes to original trial design

The trial was implemented as parallel two-arm studies as planned: two intervention arms (CPT [26] and CETA [16]) were each compared separately to a WLC arm. The two parallel studies were carried out in separate areas of Southern Iraq. The original plan for analysis was to lower the sample size requirement in each area by combining the WLC participants to comprise the comparison group for both of the intervention arms, based on reports by our partners in Iraq that populations in the two areas were similar. Over the period of the trial, it became clear that the two areas were not similar, with the CETA location experiencing higher levels of ongoing insecurity (primarily bombings). For this reason, although the three arms were implemented as designed, the controls were not combined in the analysis reported here: the CETA intervention participants are compared only to the controls from the same region (Karbala, Najaf), and the CPT intervention participants are compared to controls from the same Basra/Nassariyah area. Evidence for the differences between the two study sites is provided in the Results, with implications for the study from not combining the controls detailed in the Discussion.

### Study objectives

We carried out a rapid qualitative study before the trial using procedures described elsewhere [27, 28] to: (1) identify important problems affecting survivors of systematic violence, as perceived by survivors living in the study area; and (2) identify important tasks of men and women. In free list and key informant interviews on local problems, the most frequently mentioned mental health problems were fear, sadness and depression, anxiousness, fear of police, tenseness (easily provoked), forgetfulness, losing trust in others, and inability to sleep [29]. Because many of the responses referred to trauma-related symptoms, we decided that trauma symptoms would be the primary study outcomes, and dysfunction would be the secondary study outcome. In free lists and focus groups, we asked men about the important tasks that men do to support themselves, their family, and community; and asked the same questions of women about women. This information formed the basis of measures of dysfunction, while the qualitative data on mental health problems were used to select and adapt standard mental health instruments for local use as described below.

### Study instrument

The study instrument included the symptom section of the Harvard Trauma Questionnaire (HTQ) to assess trauma symptoms, the Hopkins Symptom Checklist for Depression and Anxiety (HSCL-25), and a separate section containing 20 frequently mentioned mental health symptoms from the qualitative study (described above) not already included in the HTQ or HSCL-25 [30–32]. Possible responses for the HTQ and HSCL-25 were how often participants experienced each symptom in the prior 2 weeks using an ordinal scale of 0 (never) to 3 (very often—i.e., five or more times per week). For example, participants were asked how often in the last 2 weeks they were ‘feeling depressed’, and the possible responses were: Never or No – score of 0; Sometimes (1–2 times a week – score of 1; Often (3–5 times a week) – score of 2; and, Very Often (more than 5 times per week) – score of 3.

During translation, one HSCL item (feeling hopeless about the future) and two HTQ items (feeling as if you don’t have a future; hopelessness) were very similar in local Arabic. We therefore included only one question on hopelessness but used it in both our trauma symptom and depression symptom scales. The final instrument included 25 HSCL symptoms and 29 HTQ symptoms.

The study instrument included locally developed dysfunction scales for men and women using a process described in [33]. These scales were derived from data from the qualitative study based on locally-described roles of men and women. Participants were asked how difficult it was for them to do each task in the prior 2 weeks on an ordinal scale of 0 (no difficulty) to 4 (unable to do the task). For example, men were asked how difficult it was for them to communicate or socialize. Women were asked how difficult it was to raise their children. In the final instrument, there were 21 items on the male dysfunction scale and 21 items on the female dysfunction scale.

Prior to the RCT, we tested the study instrument’s reliability and criterion validity among 149 survivors of systematic violence (80 men, 69 women) using a process described elsewhere [33, 34]. The re-interviews to assess test-retest reliability were carried out within 3 weeks of the first interview (the average time was seven days after the first interview). The time between the interview and re-interview was longer than usual because of disruptions caused by insecurity and holidays (Ramadan). Cronbach’s alpha scores were all greater than 0.90 indicating adequate internal reliability [35]. Pearson correlation coefficients for combined inter-rater and test-retest reliability (repeat by different interviewer) were all greater than 0.79 (range 0.799 to 0.961) suggesting good inter-rater and test-retest reliability. Criterion validity was explored by comparing the mean total scale scores of individuals diagnosed with anxiety, depression, and/or PTS respectively by a local psychiatrist to those of individuals said by a local psychiatrist to not to have any of these problems. The difference in mean total scale scores between those diagnosed by the psychiatrist with and without a condition was 14.5 (range of individual scores 0–72), 6 (range 0–51), and 25.5 (range 0–108) for depression, anxiety and trauma, respectively; all were statistically significant (*p* < .05). Among men, the difference was 20, 6, and 28 for depression, anxiety and trauma, respectively, and all differences were statistically significant (*p* < .05). Among women, the median difference was 4.5 (*p* = .488), 7 (*p* = .076), and 26.5 (*p* < .05), indicating that the scale may not adequately discriminate women with depression from those without. Overall, we concluded that criterion validity was supported for all scales except for depression among women.

Based on item analysis, the trauma symptoms on the locally validated HTQ, along with several additional local trauma symptoms (e.g., feeling that one is being watched), were used to create a trauma scale for determining eligibility for the study. A trauma scale score of 36 (the sum of each item in the scale with a maximum possible score of 105) was the symptom criterion for eligibility. We selected this cut-off score because it maximized the sensitivity and specificity based on a receiver operating characteristic curve (ROC) analysis of validity study data. The ROC analysis was based on diagnosis of PTS by the psychiatrists (dichotomous variable) and the HTQ scale score (continuous variable). Area under the curve for this analysis was 0.75 (significance = 0.000) suggesting a level of accuracy that was fair. Lending equal weight to sensitivity and specificity, 35.5 was the score at which sensitivity and 1-specificity were maximized, which we rounded up to 36.

The study instrument was translated into Iraqi Arabic using words and phrases identified during the qualitative study for symptoms. The study instrument was then back-translated to English by another translator to check for accuracy of the translation.

### Study participants

Participants were survivors of systematic violence referred to the CMHWs by physicians in the health center where they worked, from local prisoners’ associations, and through self-referral after learning of services through public service announcements or by word of mouth. Survivors were defined as persons having experienced or witnessed physical torture or militant attacks. A screening instrument was used by the CMHW both to determine a client’s eligibility for the trial and, if recruited, as their baseline assessment. The screening instrument was the same instrument used to measure the severity of symptoms experienced by participants (the dependent variable of the study). We also used the instrument to screen for eligibility for the study. The instrument had a section on dysfunction, a section on depression and anxiety symptoms, a section on trauma symptoms, a section on problems of torture survivors (identified during a qualitative study before the trial), and a section with demographic questions. A score of 36 or higher on the 29-question trauma section was the cutoff used for study eligibility based on our finding in the earlier validity study that this cutoff was optimal for discriminating those individuals diagnosed with PTSD from those without PTSD. A survivor who was 18 years of age or older and who met the symptom criterion was eligible for the trial.

Exclusion criteria included clients identified by the CMHWs as currently being psychotic and/or those who were a danger to themselves or to others. In these cases, the supervisor (a psychiatrist) was called immediately to talk to the client for possible referral to a clinic or hospital.

### Study setting

The study took place in the areas surrounding the cities of Karbala, Najaf and Hilla (CETA), and around Basra/Nassariyah (CPT) in Southern Iraq. The treatment was provided in Ministry of Health primary health care centers unless there was insufficient privacy or the client found it difficult to travel. In these situations, another mutually convenient and private place was chosen (e.g., client’s home).

### Interventions

#### Waitlist control

Waitlist control participants received monthly telephone calls from the CMHWs who enrolled them into the study to assess their safety and whether they needed referral to psychiatric care (i.e. were a danger to self or others or presented with psychosis). A safety monitoring form was used to screen for the need for referral [36]. CMHWs were instructed to check in with WLC participants but not provide any treatment. After completing their control period and second assessment, controls were retired from the trial and offered CETA or CPT.

#### Intervention: Common Elements Treatment Approach (CETA)

CETA, a transdiagnostic intervention developed by authors LM and SD, includes the following possible components: 1) encouraging participation and psychoeducation, 2) relaxation, 3) behavioral activation, 4) cognitive coping and restructuring, 5) imaginal exposure, 6) in vivo exposure, 7) safety, and 8) finishing/wrap up [16]. CMHWs were taught all components, as well as how to make decisions about selection, sequencing, and dosing (i.e. tailoring to the individual participant) based on three sources of information: 1) results from certain items on the validated study instrument, 2) client observations and statements in the assessment and early sessions, and 3) discussion with their supervisor, who in turn discussed the information with a CETA trainer [16]. CETA was designed to include approximately 8–12 weekly individual sessions of 50–60 min in length. Results from a recently completed randomized trial testing CETA with displaced Burmese on the Thai-Myanmar border showed significant reductions in depression, posttraumatic stress, dysfunction, anxiety symptoms, and aggression [37].

CETA training and supervision followed the Apprenticeship Model (see [38] for details). Briefly, CMHWs received a10-day training in CETA, and then subsequently participated in small practice groups led by two local supervisors (both psychiatrists) and completed one pilot CETA case. Throughout the trial, CMHWs participated in weekly group supervision led by local supervisors. CETA trainers, based in the United States, conducted weekly Skype calls with local supervisors to review each case and provide redirection when needed to ensure fidelity. Cultural adaptation of CETA was carried out collaboratively by the local team and US-based experts prior to and during the training process [39]. Fidelity was tracked by CMHW self-report of elements delivered, supervisor review of notes and CMHW reports, and finally by trainer review.

#### Intervention: Cognitive Processing Therapy (CPT)

Cognitive Processing Therapy (CPT) is an evidenced-based cognitive behavioral psychotherapy originally developed for treatment of PTS or PTS with comorbid depression [40, 41]. CPT combines cognitive restructuring (i.e., techniques aimed at changing extreme and/or exaggerated beliefs to be more balanced and/or realistic) with emotional processing of trauma-related content (i.e., techniques to enable clients to remember and experience the full range of emotions about their trauma). The therapy has been highly effective at reducing symptoms of PTS, depression, and anxiety across several RCTs and efficacy studies across a range of trauma exposed populations including sexual assault, child sexual abuse, domestic violence, and combat [25, 41–45]. CPT has been evaluated for use with Bosnian refugees within the United States, the majority of whom were exposed to torture, with effect sizes equal to those in the randomized clinical trials [46, 47]. In addition, CPT was highly effective at reducing symptoms of PTS, depression, and anxiety as well as decreasing dysfunction in a RCT in the Democratic Republic of Congo, a high conflict setting with low resources [48]. Given these findings, CPT appeared to be another good option to test for survivors of torture and other systematic violence in Southern Iraq.

The CPT intervention was provided using an apprenticeship model for training and supervision. The CMHWs received seven days of in-person training with expert US-based CPT trainers (DLK, KPL) based on a manual that was translated and adapted for the Southern Iraq context. Ongoing supervision was provided through a multi-tiered supervision structure: An Iraqi psychiatrist and cognitive psychologist provided direct supervision through phone or in person meetings with the CMHWs; a bilingual US-trained physician trained in CPT (GZ) provided telephone and Skype oversight and supervision to the supervisors; and this physician communicated with the US-based experts (DLK, KPL) through weekly calls for additional support and quality assurance. Cultural adaptations, described elsewhere, were made to the standard CPT treatment so as to accommodate cultural differences, better meet the needs of clients with lower levels of education, and to be easier for therapists with less training in mental health interventions to administer [26]. Participants in the intervention group attended individual therapy sessions with CMHWs. Therapy was 12 sessions, usually 1 week apart.

### Outcomes

The primary outcome was trauma symptoms, assessed by a trauma scale score representing the mean of the scores given to responses on the locally-validated HTQ. The secondary outcome was dysfunction, assessed by mean item scores for the gender-specific items on the locally-developed dysfunction scale. Anxiety and depression were assessed using the mean item score on the locally-validated HSCL-25. None of the local items derived from the qualitative study are included in the outcome scores for trauma, depression or anxiety; the local items were used solely to screen clients for eligibility into the study.

### Sample size

Our sample size calculation of *N* = 150 per arm provides 80 % power to detect a moderate effect size of 0.50 (Cohen’s d), with an estimated loss of 25 % due to the authors’ experience with dropout in similar settings, the additional expected dropout due to insecurity, and a moderate design effect of 1.5 given authors’ experience and a lack of other studies in the region.

### Randomization

A randomization list was generated separately for each CMHW by study investigators. This list included 20 sequential participant identification numbers. The assignment was generated using a random number generator in Excel, with a 2 to 1 probability of assignment to the intervention vs. the waitlist. A piece of paper indicating the treatment assignment (intervention or waitlist) was stapled directly to the back of the study consent forms that were pre-numbered with the participant identification number. This paper could only be read if removed from the consent form.

Each potential study participant presenting to the CMHW with a request for mental health services was interviewed using the study instrument. After identifying a client as eligible for the study, and after obtaining their informed consent to participate, the CMHW detached the study assignment paper stapled to the consent form. The study investigators and supervisors maintained a master list for each CMHW that indicated the sequence and appropriate treatment status (intervention/WLC) for each participant to enable checking fidelity to the randomization model.

To avoid a difference between intervention and WLC participants in the time between baseline and follow-up assessments, we matched controls with an intervention participant who was enrolled into the study about the same time (within a few days to a week). When an intervention participant—with an identified control match—finished therapy, we arranged to interview both as close together in time as possible. The matching was done after the trial began but before any follow-up interviews were carried out.

### Blinding

Baseline assessments were conducted by CMHWs as part of the recruitment process prior to randomization and who were therefore blind to the assignment of study participants to intervention or WLC. These CMHWs treated those persons they had recruited who were randomly assigned to treatment. Therefore, to maintain blinding, follow-up interviews were done by a different CMHW than the one who recruited the participant so they were unaware of the participant’s assignment. The supervisors and the study participants were not blind to the treatment condition.

### Statistical methods

All analyses were conducted using Stata 12 [49]. Multiple imputation techniques were used to account for missingness at the item level and the participant level. Missing data, including information about participants who were lost to follow-up, were imputed using STATA’s chained equations command for multiple imputation (MI) using Rubin’s rules for pooling data [50, 51]. Missing at random (MAR) was assumed for the imputation model due to the low rate of missing follow up interviews. There were two clients in the CPT trial with more than 40 % of the items missing in their baseline assessment of anxiety. There were also two clients in the CPT trial with more than 40 % of items missing in their baseline assessment of depression. When looking at individual items, there were no items with more than 5 % of total responses missing in the baseline data. Nine CPT clients and three CETA clients had no follow up scores due to not receiving follow up or lost records. Among those who had follow up scores recorded, there was one client in the CPT trial who had more than 40 % of the items missing in the function scale and one client in the CETA trial who had more than 40 % of the items missing in the trauma scale. When looking at individual items, there were no items with more than 5 % of total responses missing in the follow up data among those with recorded follow up.

Missing data on demographic variables were imputed based on all other demographic variables, the counselor id-number and treatment status. We then imputed missing baseline and follow-up scores using all of the variables in the dataset including treatment or control status. CETA and CPT participants were imputed separately. Average scores for all outcome variables were then calculated using 11 imputed datasets. We did not do any data transformations. All final outcome models were run using the 11 imputed datasets.

For each outcome measure, we calculated the net difference in mean score between intake to follow-up and between intervention and control participants, along with the effect size of the intervention. Treatment effects were determined using longitudinal, multilevel models with CMHW and client as random effects, and a time by group interaction with robust variance estimation, to test for the net difference in mean score for each outcome between the baseline and follow up interview. We decided to use the CMHW and client as random instead of fixed effects based on the results of the Hausman test with significance set at *p <* 0.05 [52]. The significance level for treatment effects was *p* = 0.05, two-tailed, expressed as a 95 % confidence interval. Cohen’s d was used to calculate the size of the effect over and above the change experienced by the WLC participants. Cohen’s d was calculated using the difference in differences in outcomes between groups as the numerator, and the pooled standard deviation at baseline as the denominator [53]. The following interpretation was used for effect size: 0 = no effect; 0.2 = small effect; 0.4 = moderate effect; 0.8 + = large effect [54]. All analyses used the full intention-to-treat (ITT) sample.

### Sensitivity analysis

In the primary analysis, the regression models were not adjusted with added covariates such as age, gender, educational level. The dependent variable (mean scale score) was modeled against only two independent variables: intervention status (intervention or control) and time (time 1 or time 2). We assumed that the randomization process was sufficient to make the intervention and control groups equal for the main analysis (the unadjusted model). As a check, we did a sensitivity analysis that adjusted the regression model with additional independent variables (the adjusted model) such as age, gender, education status, working status. If the findings are similar, this provides more confidence that the randomization process sufficiently equalized the intervention and control groups. This model included variables that differed at baseline between intervention and control or were associated with changes in outcome measures defined as *p* < 0.10. In the final adjusted model all variables used for adjustment were also centered at their means.

## Results

### Participant flow

Recruitment was active April 2011 through January 2012 and the intervention period extended to April 2012. The trial ended two months beyond the planned trial period due to additional funding available and a slower enrollment rate than planned.

#### Intervention: Common Elements Treatment Approach (CETA)

**Fig. 1:**
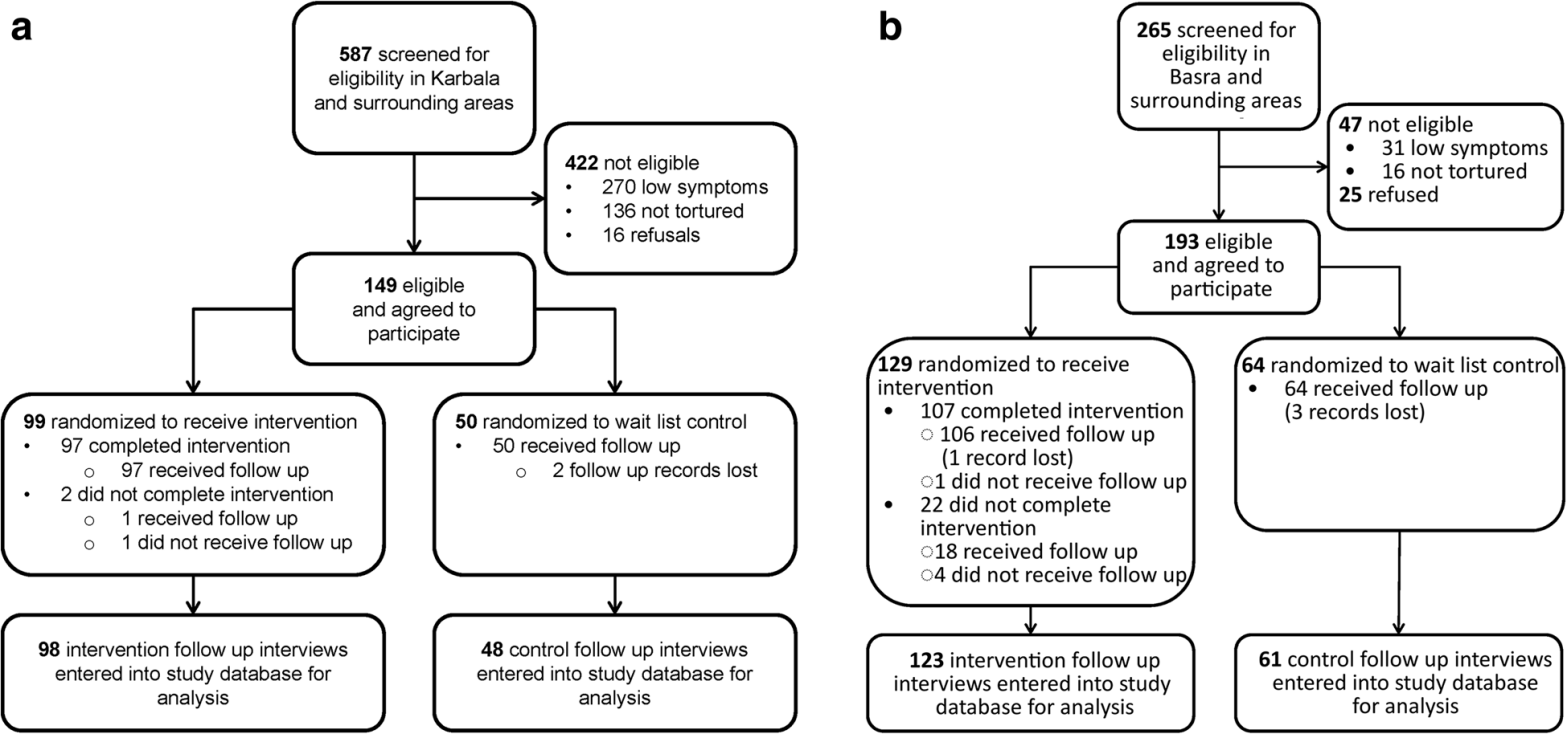
**a** Potential participants in CETA arm = Clients of community mental health workers (CMHWs) trained in CETA and the trial protocol. RCT participants = Clients of CMHWs who met the eligibility criteria of (1) exposure to torture or systematic violence, (2) meeting the cut-off score for symptoms of trauma, and (3) providing informed consent and agreeing to participate in the trial. **b** Potential participants in CPT arm = Clients of community mental health workers (CMHWs) trained in CPT and the trial protocol. RCT participants = Clients of CMHWs who met the eligibility criteria of (1) exposure to torture or systematic violence, (2) meeting the cut-off score for symptoms of trauma, and (3) providing informed consent and agreeing to participate in the trial

Five hundred and eighty seven adults were screened for eligibility, 165 (28 %) met the inclusion criteria, and 149 (90 % of those eligible) agreed to participate (Fig. 1a). Ninety-nine (66 % of 149) were randomized to receive CETA and 50 (34 %) to WLC. Follow up data were collected for 146 of the 149 (98 %) participants. Of the 99 persons enrolled in the CETA arm, 97 participants (98 %) completed therapy; all 97 were reassessed at follow up. Of the two participants in the CETA arm that dropped out, one completed the follow up interview. Of the 50 controls, all completed a follow up interview, but the interview forms for two controls were lost. All 99 intervention and 50 control participants were included in the final analysis under an intention to treat approach.

The mean number of days from intake to the second assessment was 230 for intervention clients and 249 for controls. The mean time from the end of treatment until the follow up interview among CETA participants was 3.5 months, or 135 days (inter-quartile range 89 to 176 days). CMHWs provided on average 9.94 CETA sessions (range 7 to 14). Because of the inclusion criteria of traumatic exposure and symptoms, all participants received the following components: (1) encouraging participation & psychoeducation; (2) cognitive coping/restructuring; (3) imaginal exposure; (4) safety; and, (5) finishing/wrap-up [16]. Some participants received the additional components for comorbid symptoms: 26 received relaxation, 12 received behavioral activation, and one received In Vivo exposure.

#### Intervention: Cognitive Processing Therapy (CPT)

Of the 265 persons screened in the CPT area, 218 (82 %) were eligible for enrollment into the study (Fig. 1b). Of the 218 participants eligible for the study, 193 persons aged 18 to 70 agreed to participate and were randomized to intervention or a wait-list control. Cognitive processing therapy (CPT) was provided to 129 persons in the intervention group but not the 64 persons in the wait-list control group. Twenty-two persons (all in the intervention group) dropped out of the study. One-Hundred, eighty-eight persons completed follow up interviews (106 intervention completers, 18 intervention drop outs, all 64 controls). Follow up forms from one of the intervention completers and three of the controls were unable to be located. The mean number of days from intake to the second assessment was 224 days for intervention clients and 230 days for controls. The mean time from the end of treatment until the follow up interview among intervention participants was 4.5 months, or 130 days (range 43 to 376 days). All 124 intervention and 64 control participants were included in the final analysis under an intention to treat approach.

#### Adverse events

One client attempted suicide after doing the intake and the first therapy session. The family refused to have the client admitted to the hospital because of stigma but the client was referred to a psychiatrist. The local supervisor learned about the case five days after intake during the supervision meeting. The therapist had failed to immediately notify the supervisor per protocol. We learned that the client was a relative of the therapist (not appropriate) and the therapist reported a conflict between providing care and concerns about stigma to his family. The supervisor was unable to follow up with the psychiatrist due to requests for privacy on the part of family of the client. This client was dropped from the study.

Another client was hospitalized with severe depression, received therapy in the hospital, was discharged after feeling better, but refused to return to the study. One patient died from a heart attack but this has no apparent relationship to participation in the study.

### Baseline demographic and clinical characteristics

#### Intervention: Common Elements Treatment Approach (CETA)

There appear to be some differences between intervention and control groups in age (controls were older), percent married (fewer controls were single) and percent with disability (fewer controls were disabled) (Table 1). We did not identify any apparent differences between intervention and control clients in trauma, anxiety, depression, dysfunction, gender, percent working, percent with education, or number of children.

**Table 1:** Baseline characteristics of intent-to-treat sample - CETA

	Group		
Category	Subcategory	Intervention	Control
N		99	50
Sex, N (%)	Male	67 (67.7)	36 (72.0)
	Female	32 (32.3)	14 (28.0)
Age, mean (SD)		41.6 (11.3)	45.16 (11.1)
Children, mean (SD)		2.30 (2.0)	2.34 (2.0)
Marital status, N (%)	Single	13 (13.1)	2 (4.0)
	Married	73 (73.7)	44 (88.0)
	Widowed	10 (10.1)	3 (6.0)
	Divorced,	3 (3.0)	1 (2.0)
Working status, N (%)	Not working	36 (36.4)	17 (34.0)
	Irregular or daily	25 (25.3)	8 (16.0)
	Regular or stable	34 (34.3)	20 (40.0)
	Self-employed	4 (4.0)	5 (10.0)
Education, N (%)	None	15 (15.0)	3 (6.0)
	Primary	30 (30.3)	21 (42.0)
	Secondary	33 (33.3)	12 (24.0)
	Institutional degree	16 (16.2)	6 (12.0)
	Bachelor’s degree or higher	5 (5.1)	8 (16.0)
Disability, N (%)		13 (13.1)	1 (2.0)
Mental health symptoms scales, mean (SD)	Harvard Trauma score	1.3 (0.2)	1.3 (0.3)
	HSCL anxiety score	1.4 (0.4)	1.3 (0.4)
	HSCL depression score	1.3 (0.4)	1.3 (0.4)
Dysfunction scales, mean (SD)	Male dysfunction score	1.6 (0.6)	1.6 (0.6)
	Female dysfunction score	1.4 (0.6)	1.5 (0.3)

*SD* standard deviation

#### Intervention: Cognitive Processing Therapy (CPT)

There were no apparent differences between intervention and control clients in demographic characteristics (Table 2).

**Table 2:** Baseline characteristics of intent-to-treat sample – CPT

	Group		
Category	Subcategory	Intervention	Control
N		129	64
Sex, N (%)	Male	87 (67.4)	40 (62.5)
	Female	42 (32.6)	24 (37.5)
Age, mean (SD)		40 (12.3)	41 (9.5)
Children, mean (SD)		2.2 (2.1)	2.7 (2.1)
Marital status, N (%)	Single	20 (15.5)	4 (6.3)
	Married	95 (73.6)	50 (78.1)
	Widowed	4 (3.1)	4 (6.3)
	Divorced	10 (7.8)	6 (9.4)
Working status, N (%)	Not working	55 (42.6)	24 (37.5)
	Irregular or daily	19 (14.7)	11 (17.2)
	Regular or stable	46 (35.7)	25 (39.1)
	Self-employed	9 (7.0)	4 (6.3)
Education, N (%)	None	20 (15.5)	13 (20.3)
	Primary	48 (37.2)	32 (50.0)
	Secondary	29 (22.5)	12 (18.8)
	Institutional degree	18 (14.0)	4 (6.3)
	Bachelor’s degree or higher	14 (10.9)	3 (4.7)
Disability, N (%)	Yes	9 (7.0)	5 (7.8)
	No	120 (93.0)	59 (92.2)
Mental health symptoms scales, mean (SD)	Harvard Trauma score	1.5 (0.4)	1.5 (0.4)
	HSCL anxiety score	1.5 (0.5)	1.6 (0.6)
	HSCL depression score	1.6 (0.5)	1.6 (0.6)
Dysfunction scales, mean (SD)	Male dysfunction score	1.1 (0.7)	1.1 (0.7)
	Female dysfunction score	1.4 (0.8)	1.7 (0.9)

*SD* standard deviation

### Differences in the amount of change in symptom scores between CETA vs. CPT control participants

Control participants living in the CPT intervention area improved significantly more than the controls in the CETA intervention area. The mean trauma symptom scores of CPT controls (on a scale of 0–3) dropped 0.59 more than those of the CETA controls (a change of −0.92 vs. -0.32, respectively) see Table 3. The effect size (Cohen’s d) of this difference is large (1.54). The mean anxiety symptom scores of CPT controls dropped 0.57 more than those of the CETA controls (a change of −0.88 vs. -0.31, respectively, effect size = 1.1). And, the mean depression symptom scores of CPT controls dropped 0.67 more than those of the CETA controls (a change of −0.90 vs. -0.23, respectively; effect size = 1.3).

**Table 3:** Change in scale scores comparing CPT control to CETA control participants (*N* = 114)

	CETA Controls (*N* = 50)		CPT Controls (*N* = 64)		Net Effect^a^		Effect Size
	Score	95 % CI	Score	95 % CI	Score	95 % CI	
Harvard Trauma Scale (ICC = 0.30)							
Baseline	1.27	1.19 to 1.35	1.57	1.42 to 1.71			
Follow Up	0.95	0.70 to 1.20	0.65	0.47 to 0.83			
Pre-post change	−0.32	−0.57 to −0.07	−0.92	−1.09 to −0.74	−0.60	−0.90 to −0.30	1.55
HSCL Anxiety Scale (ICC = 0.22)							
Baseline	1.31	1.16 to 1.46	1.60	1.42 to 1.77			
Follow Up	1.00	0.76 to 1.25	0.72	0.55 to 0.89			
Pre-post change	−0.31	−0.53 to −0.09	−0.88	−1.09 to −0.67	−0.57	−0.88 to −0.27	1.11
HSCL Depression Scale (ICC = 0.38)							
Baseline	1.20	1.08 to 1.33	1.62	1.39 to 1.85			
Follow Up	0.97	0.72 to 1.22	0.72	0.53 to 0.92			
Pre-post change	−0.23	−0.45 to −0.02	−0.90	−1.10 to −0.70	−0.66	−1.00 to −0.37	1.28
Function Scale (ICC = 0.55)							
Baseline	1.58	1.38 to 1.79	1.42	1.04 to 1.80			
Follow Up	1.34	1.08 to 1.59	0.93	0.63 to 1.22			
Pre-post change	−0.25	−0.50 to 0.00	−0.49	−0.71 to −0.27	−0.24	−0.58 to 0.09	0.34

*SD* standard deviation, *CI* confidence interval, *ICC* intraclass correlation coefficient, *HSCL* Hopkins Symptom Checklist

^a^ Model-estimated difference at post-test with CMHW as a random intercept

These differences across the two study areas, in our opinion, precluded our original plan to combine the control groups across the two study areas for analyses. Instead, the CETA intervention participants are compared only to the controls from the same region (Karbala, Najaf), and the CPT intervention participants are compared to controls from the same Basra/Nassariyah area.

### Outcomes and estimation

#### Intervention: Common Elements Treatment Approach (CETA)

CETA showed statistically significant improvements over WLC for all outcomes. Mean symptom scores decreased by 0.59, 0.68, 0.67, and 0.50 more in the intervention group for trauma, anxiety, and depression (range 0 to 3), and dysfunction (range 0 to 4), respectively (Table 4). Effect sizes were 2.40 for trauma symptoms, 1.60 for anxiety, 1.82 for depression, and 0.88 for dysfunction (Table 4).

**Table 4:** Change in scale scores comparing CETA intervention to control participants (*N* = 149)

	CETA Intervention (*N* = 99)		CETA Controls (*N* = 50)		Net Effect^a^		Effect Size
	Score	95 % CI	Score	95 % CI	Score	95 % CI	
Harvard Trauma Scale (ICC = 0.12)							
Baseline	1.29	1.24 to 1.35	1.28	1.19 to 1.36			
Follow Up	0.39	0.27 to 0.50	0.96	0.70 to 1.21			
Pre-post change	−0.91	−1.06 to −0.76	−0.32	−0.57 to −0.06	−0.59	−0.76 to −0.42	2.40
HSCL Anxiety Scale (ICC = 0.06)							
Baseline	1.40	1.25 to 1.55	1.33	1.18 to 1.48			
Follow Up	0.41	0.33 to 0.49	1.02	0.78 to 1.27			
Pre-post change	−0.99	−1.20 to −0.78	−0.31	−0.53 to −0.08	−0.68	−0.85 to −0.52	1.60
HSCL Depression Scale (ICC = 0.05)							
Baseline	1.32	1.21 to 1.43	1.24	1.13 to 1.35			
Follow Up	0.42	0.31 to 0.53	1.01	0.75 to 1.26			
Pre-post change	−0.90	−1.11 to −0.70	−0.23	−0.46 to −0.01	−0.67	−0.80 to −0.53	1.82
Function Scale (ICC = 0.20)							
Baseline	1.55	1.36 to 1.73	1.62	1.42 to 1.83			
Follow Up	0.80	0.59 to 1.00	1.38	1.13 to 1.63			
Pre-post change	−0.75	−0.95 to −0.55	−0.25	−0.50 to 0.01	−0.50	−0.73 to −0.27	0.88

*SD* standard deviation, *CI* confidence interval, *ICC* intraclass correlation coefficient, *HSCL* Hopkins Symptom Checklist

^a^ Model-estimated difference at post-test with CMHW as a random intercept

#### Intervention: Cognitive Processing Therapy (CPT)

CPT showed statistically significant improvements over WLC for trauma and depression symptoms. Mean symptom scores decreased by 0.16 and 0.22 more in the intervention group for trauma and depression (range 0 to 3), respectively (Table 5). Effect sizes were moderate (0.41 for trauma and 0.40 for depression). The treatment effects for anxiety and function were small to null, and the difference between intervention and WLC was not statistically significant at the .05 level. Mean symptom scores for anxiety (range 0 to 3) and dysfunction (range 0 to 4) decreased by 0.14 and 0.05, respectively.

**Table 5:** Change in scale scores comparing CPT intervention to control participants (*N* = 193)

	CPT Intervention (*N* = 154)		CPT Controls (*N* = 64)		Net Effect^a^		Effect Size
	Score	95 % CI	Score	95 % CI	Score	95 % CI	
Harvard Trauma Scale (ICC = 0.24)							
Baseline	1.53	1.40 to 1.67	1.55	1.40 to 1.69			
Follow Up	0.45	0.33 to 0.57	0.63	0.45 to 0.81			
Pre-post change	−1.08	−1.26 to −0.90	−0.92	−1.09 to −0.74	−0.16	−0.31 to −0.02	0.41
HSCL Anxiety Scale (ICC = 0.13)							
Baseline	1.53	1.37 to 1.68	1.58	1.41 to 1.75			
Follow Up	0.50	0.41 to 0.60	0.70	0.53 to 0.88			
Pre-post change	−1.02	−1.21 to −0.83	−0.88	−1.09 to −0.66	−0.14	−0.32 to 0.03	0.27
HSCL Depression Scale (ICC = 0.24)							
Baseline	1.60	1.41 to 1.79	1.60	1.38 to 1.83			
Follow Up	0.48	0.35 to 0.62	0.70	0.51 to 0.90			
Pre-post change	−1.11	−1.36 to −0.87	−0.90	−1.10 to −0.70	−0.22	−0.38 to −0.05	0.40
Function Scale (ICC = 0.51)							
Baseline	1.29	1.00 to 1.59	1.38	1.02 to 1.74			
Follow Up	0.75	0.55 to 0.94	0.89	0.62 to 1.16			
Pre-post change	−0.54	−0.75 to −0.33	−0.49	−0.72 to −0.27	−0.05	−0.25 to 0.15	0.07

*SD* standard deviation, *CI* confidence interval, *ICC* intraclass correlation coefficient, *HSCL* Hopkins Symptom Checklist

^a^ Model-estimated difference at post-test with CMHW as a random intercept

#### Sensitivity analysis

The adjusted-model results were almost identical to the results of the unadjusted model for both CETA and CPT. Mean adjusted CETA symptom scores decreased by 0.59, 0.66, 0.65, and 0.49 more in the intervention group for trauma, anxiety, and depression (range 0 to 3), and dysfunction (range 0 to 4), respectively, after adjusting for baseline scores and other factors. Effect sizes were 2.38 for trauma symptoms, 1.56 for anxiety, 1.78 for depression, and 0.87 for dysfunction. Mean adjusted CPT symptom scores decreased by 0.17 and 0.22 more in the intervention group for trauma and depression (range 0 to 3), respectively, after adjusting for baseline scores and other factors. Effect sizes were moderate for trauma (0.42) and for depression (0.40). Mean symptom scores for anxiety (range 0 to 3) and dysfunction (range 0 to 4) decreased by 0.15 and 0.05, respectively, were not statistically significant, and the effect sizes were small to null.

## Discussion

Participants receiving CETA showed large and statistically significant improvements in trauma, depression, anxiety, and dysfunction compared to wait-list control participants and as compared to many other studies of CBT. For example, Rahman and colleagues [54]—in a study in rural Pakistan that included married women (aged 16–45 years) in their third trimester of pregnancy with perinatal depression, using providers who were primary health workers—found effect sizes for CBT of 0.70–0.80 for depression, disability and functioning. The effect size for interpersonal psychotherapy (IPT) in Southern Uganda was 1.87 for depression among adults [55]. Effect sizes of 1.16 for depression, 1.19 for PTS, 0.79 for anxiety, 0.60 for dysfunction, and 0.58 for aggression were found for an RCT of CETA among Burmese living in Thailand [37]. Participants receiving CPT in this trial experienced moderate improvements in trauma and depression, but small to no improvement in anxiety and function, relative to WLC, in part due to larger improvements in the WLC for the CPT regions. In addition to the CPT trial reported here, a trial of CPT in Northern Iraq had medium effect sizes on depression or functioning and large effect sizes on PTSD and traumatic grief [25]. In contrast, a trial of CPT in the Democratic Republic of Congo found large effect sizes (combined depression/anxiety ES = 1.8; post-traumatic stress ES = 1.4) [48].

We investigated possible reasons for the relatively large effect sizes for CETA, including differences in the treatments themselves, the supervision structure, context for the trials, counselor differences, and treatment completion rates. First, the therapy components and structure were different between CETA and CPT. The number and sequence of the components provided in CPT were relatively fixed, compared to CETA which was designed for adaptation to each participant based on their presenting symptoms and experiences. Supervision of CETA CMHWs was provided by two Iraqi psychiatrists through weekly in person meetings and these psychiatrists communicated directly with the US-based CETA experts (LM, SD) through weekly calls for additional support and quality assurance. Supervision for CMHWs providing CPT; however, was provided through a multi-tiered supervision structure, in which the CPT experts did not communicate directly with the area CPT supervisors, due to language issues (the area supervisors did not speak English), but instead communicated with area supervisors through an intermediary supervisor who was bilingual and who was not located in Southern Iraq (GZ).

We considered the differences in context between the two areas in which the interventions were tested. Trauma symptoms among controls in the CETA area improved less than the controls in the CPT area but more than the controls in a prior RCT testing CPT in Northern Iraq. Controls living in the CPT area in this trial may have improved more than the CETA controls because the CPT controls experienced a better security situation (fewer bombings) and lived closer to a large urban area (Basra). It is possible that CPT controls in this trial were more likely than CETA controls to access other mental health services or be able to travel to participate in social activities that helped them cope with their mental health problems. We considered counselor difference in education in the two trials. The education level of CMHWs was similar across the two study areas was discounted as a factor explaining the relative difference in effect sizes; however, other unmeasured CMHW characteristics could play a role in the differences.

Finally, completion rates for participants receiving CETA were very high (98 %). The completion rates for the CPT trial reported here, and the CPT trial in Northern Iraq was 89 and 84 %, respectively. We investigated possible reasons for the high completion rate compared to other trials—including cultural differences, a stable population in this trial site, and CMHW skills and practices—by interviewing the CETA trainers and supervisors. The most likely explanation for the difference in completion rates was location of where CMHWs provided therapy. In contrast to the CMHWs in the other Iraq CPT areas, CMHWs in in the CETA area more frequently provided therapy in the homes of clients (particularly important for women in this setting) or in other mutually convenient places upon client request. In addition, the CETA transdiagnostic approach allowed for individual tailoring in treatment elements delivered and number of sessions which might have resulted in greater engagement and client completion rates (e.g., some clients could finish treatment in fewer sessions).

We also explored the possibility of misconduct on the part of the research team working in the CETA area (CMHWs, supervisors, interviewers doing the follow up interviews) as possible causes of the large difference in effect sizes by examining the distributions of the participants’ pre- and post-intervention scores by CMHW and by the interviewer who conducted the blinded follow up interview. We looked for evidence of efforts to artificially create a large effect size by checking if there appeared to be any systematic attempt to lower the follow up scores of intervention clients compared to controls. Data distributions did not suggest misconduct by the CMHWs or follow-up interviewers (see Supporting Information below and Additional file 1: Figures S1 and S2).

This study (along with the trial of CPT in Northern Iraq [25]) provides evidence that CETA and CPT can be provided in Iraq by non-specialists based in primary health care centers, under supervision and mentorship by trained supervisors. Mental health treatment tasks were shared, with trained mental health professionals providing supervision and mentoring, and minimally trained (but supervised) non-specialists providing direct therapy to persons in need.

CETA was found to be a highly effective intervention across symptoms in the trial setting. Similar transdiagnostic interventions to CETA are showing promise in high-income countries (HIC) for the treatment of comorbidity by individualizing treatment [22, 23]. However, in HIC trials, highly trained mental health professionals, or the researchers themselves, have made most decisions about which components should be provided and the dose and sequencing of components. In this study the CETA CMHWs made those decisions on their own in most cases, with monitoring by local supervisors and the CETA trainers. By individualizing treatment, including adding components as needed (such as behavioral activation), CMHWs were able to use CETA to address symptoms of depression and anxiety as well as trauma.

### Generalizability

This trial evaluated the impact of two therapies for use in LMIC, among mostly rural survivors of systematic violence (torture and militant attacks) in Southern Iraq who were experiencing trauma symptoms at enrollment. Most of these participants also experienced symptoms of depression and anxiety, as well as dysfunction. We do not know whether CETA and CPT would produce similar results among Iraqis that did not experience systematic violence, or non-trauma exposed individuals with the same mental health problems. We do not know how well CETA performs in other cultures except for one similar trial among Burmese trauma survivors in Thailand in which the authors found CETA to be effective for the same problems as in Iraq [37].

When assessing generalizability, issues related to implementation issues should also be considered. For example, in both the CETA and CPT sites, CMHWs had other duties in and outside of the clinic (e.g., vaccination campaigns). In addition, clients traveled occasionally resulting in missed sessions some weeks. As designed, supervisors were expected to meet weekly with CMHWs to review cases, provide guidance, practice treatment elements for upcoming sessions, and identify issues for discussion with experts. In practice, security issues (bombings, checkpoints and road closures) and problems with the phone or internet sometimes interfered with supervision schedules in both conditions, and supervision phone calls did not always occur weekly. In addition, supervisors had other duties (e.g., academic responsibilities) that occasionally led to scheduled supervision sessions being missed. Supervision occurred more irregularly in the CPT arm, as one supervisor was often traveling and/or not available to make the scheduled calls with the local overseeing physician (GZ) or the experts, which led to difficulty for the CPT experts in having a clear sense of the treatment implementation (e.g., specific case information [client progress, CMHW fidelity] was frequently unavailable to the experts); thus evaluating fidelity and providing redirection was often challenging. As the overseeing physician was also off-site, this added an additional layer of complexity to monitoring implementation. This suggests that the supervision structures could be critical to effectiveness and generalizability.

### Contribution to the literature

To our knowledge, this trial is one of only two of non-drug mental health interventions completed so far in Iraq and perhaps the Middle East (a second trial was conducted by our research group in the Kurdish area of Iraq [25]). In addition, this trial is one of only three testing CPT in a low resource environment and one of only two testing delivery of a transdiagnostic intervention (CETA), with both interventions using a task-sharing approach in a low resource setting.

### Limitations

This study did not evaluate longitudinal effects of CETA and CPT. Although the follow-up was, on average, more than four months post-treatment, additional post-intervention assessments of six to 12 months after treatment would be more informative. While post-intervention interviewers were blinded to participants’ allocation, participants and counselors were not. It is possible that the post-intervention interviewer could, through questioning of his/her own (although instructed not to), gather sufficient information to learn the assignment and intentionally, or not, differentially assess outcomes. Due to security reasons, research staff members from the U.S. were not able to visit any sites, limiting the team to remote oversight although internet and phone allowed us to be in weekly frequent contact with supervisors, counselors and research staff. This study did not have the capacity to investigate the mechanism of action (which specific elements, sequence, or dosing were predictive of changes). There was no placebo attention-control condition and wait-list-control participants did not meet in person with the CMHWs on an ongoing basis. Therefore, it is not clear how much of the intervention effects are due to the meetings with the counselor regardless of the content. Our study instrument lacked evidence supporting criterion validity in women for depression.

We chose to compare intervention participants to wait list controls rather than an active control group. We understand that active controls are preferable where there is an existing standard of treatment that is known to be effective. We could find no prior research on the effectiveness of any mental health intervention among this population, nor was there a mental health or psychosocial intervention in common use. We did consider having an active control group consisting of clients meeting weekly with counselors who did not have training in CETA or CPT, to account for the effect of weekly meetings. We rejected this as being unstandardized in terms of approach and content. In other words, we would not be able to say to what we were comparing the intervention. Only by having a true control group and subtracting the change from that group did we feel that the basic question could be answered of whether either intervention was effective, ineffective, or even harmful. In addition, standardization of therapy among active controls would have required developing specific materials and training and supervising additional workers. Given the lack of literature supporting non-specific interventions it would have been difficult to justify this to our partners.

## Conclusions and future directions

We found a common elements or “transdiagnostic” mental health intervention (CETA) to be very effective, and CPT to be moderately effective, when provided by CMHWs for survivors of systematic violence in Southern Iraq compared to wait-list controls. The role of the scarce mental health professionals (psychiatrists or psychologists) shifting from treating a few people to supervising the treatment of many persons through the CMHWs is supported as CMHWs were able to learn and provide both interventions with fidelity. This approach to task sharing is supported by other literature as a sound option for providing sustainable, accessible, and effective services for multiple mental health problems at scale where there are few professionals [56, 57]. In addition, the CETA approach allows non-specialized health workers to select from a range of evidence-based therapy elements to tailor treatment to address a variety of common and comorbid mental health problems in a primary health care setting. However, we cannot conclude that one intervention is better than the other. The CETA and CPT interventions were carried out in different settings, run as independent parallel trials, with different supervision procedures, making comparisons problematic. Future research should include more trials of both treatment approaches in diverse settings, with broader inclusion criteria, an active control, by independent researchers, and with a longer follow up period.

## Additional files

### Additional information about development of the study instrument

The validity study described in the article identified large and statistically significant differences among all the scale scores between psychiatrist-diagnosed cases and non-cases, except for depression among women [34]. This finding was mainly due to a higher mean depression score among female non-cases compared with male non-cases. Despite failing to support criterion validity for depression among women we decided to use the depression scale in the study instrument because of its solid performance on the other validity and reliability measures.

Based on item analysis, six local problems were added to the adapted HTQ items to form the trauma scale used to assess trial eligibility: loss of self-confidence; violence with the family; problems with social relationships; tense (easily provoked); losing trust in others; and, having the feeling that I am watched. In addition, the following item was dropped from the HTQ during validation based on item analysis: hopelessness. No items were dropped from the HSCL-25, which constituted the depression and anxiety scales.

The dysfunction scales were developed from frequently mentioned responses to questions in the qualitative study about the most important tasks for men/women to do to take of themselves, their family and the community.

### Analysis of CETA baseline and final trauma scores and change in scores

Two figures (Additional file 1: Figures S1 and S2) are provided as supporting information to statements in the discussion that although the CETA trial had a very large effect size for trauma, it is unlikely that this is due to fraudulent interviewing or recording by either the client’s CMHW or the person who conducted the follow up interview. The figures show the distribution of participants’ baseline and follow up scores organized by CMHW (Additional file 1: Figure S1) and by follow up interviewer (Additional file 1: Figure S2). Additional file 1:
**Figure S1.** Baseline and follow up trauma scores for intervention and control clients by CMHW. The plot on the left shows the baseline and final trauma symptom scores of controls and the plot on the right shows the symptom scores of clients who received CETA. Baseline scores are highlighted with blue symbols; follow up scores have black symbols. Grey lines connect the baseline with the follow up score and the length reflects the amount of change. Red text indicates the CMHW who enrolled the control clients and who provided therapy to the CETA clients. The distribution of scores does not suggest any systematic manipulation of scores in a way to create the large effect size found in this trial. Baseline symptom scores vary across clients, and vary within the clients of the CMHWs. Follow up scores also vary across and within the clients of CMHWs, with a few exceptions. Where a particular CMHW has a group of intervention clients with similar follow up scores (e.g., 202, 211), the controls have similar follow up scores to that CMHW’s intervention clients. If there was an effort by these two CMHWs to artificially create a large effect size, we would expect the opposite pattern: intervention clients would have very different and lower follow up scores than controls. **Figure S2.** Baseline and follow up trauma scores for intervention and control clients by follow up interviewer. The plot on the left shows the baseline and final trauma symptom scores of controls and the plot on the right shows the scores of clients who received CETA. Baseline symptom scores are highlighted with blue symbols; follow up scores have black symbols. Grey lines connect the baseline with the follow up score and show the amount of change between the two time points. Green text indicates the interviewer who conducted the follow up interview for a particular client. As in Figure S1, the distribution of scores does not suggest any systematic manipulation of scores in a way to create the large effect size found in this trial. Follow up scores vary across clients. Follow up scores vary within the clients who met with a particular follow up interviewer, with a few exceptions. Where a particular interviewer has a group of intervention clients with similar follow up scores (e.g., 202, 211), the controls also have similar follow up scores to the intervention clients. If there was an effort by these two interviewers (who interviewed each other’s clients) to artificially create a large effect size, we would expect intervention clients to have much lower follow up scores than controls. (ZIP 296 kb)

## Abbreviations

CBI: Components based intervention (original name given to CETA)
CETA: Common elements treatment approach
CMHW: Community health worker
CBT: Cognitive behavioral therapy
CPT: Cognitive processing therapy
EBT: Evidence-based treatment
HTQ: Harvard Trauma Questionnaire
HSCL: Hopkins Symptom Checklist for Depression and Anxiety
LMIC: Low and middle income country
NET: Narrative exposure therapy
RCT: Randomized control trial
PTS: Posttraumatic stress
PTSD: Posttraumatic stress disorder
TT: Testimony therapy
WLC: Waitlist control
