# Supplementary figures and images for: Community-based mental health treatments for survivors of torture and militant attacks in Southern Iraq: a randomized control trial
Source: BMC Psychiatry. 2015 Oct 14;15:249. doi: 10.1186/s12888-015-0622-7 (PMC4605204; doi:10.1186/s12888-015-0622-7)

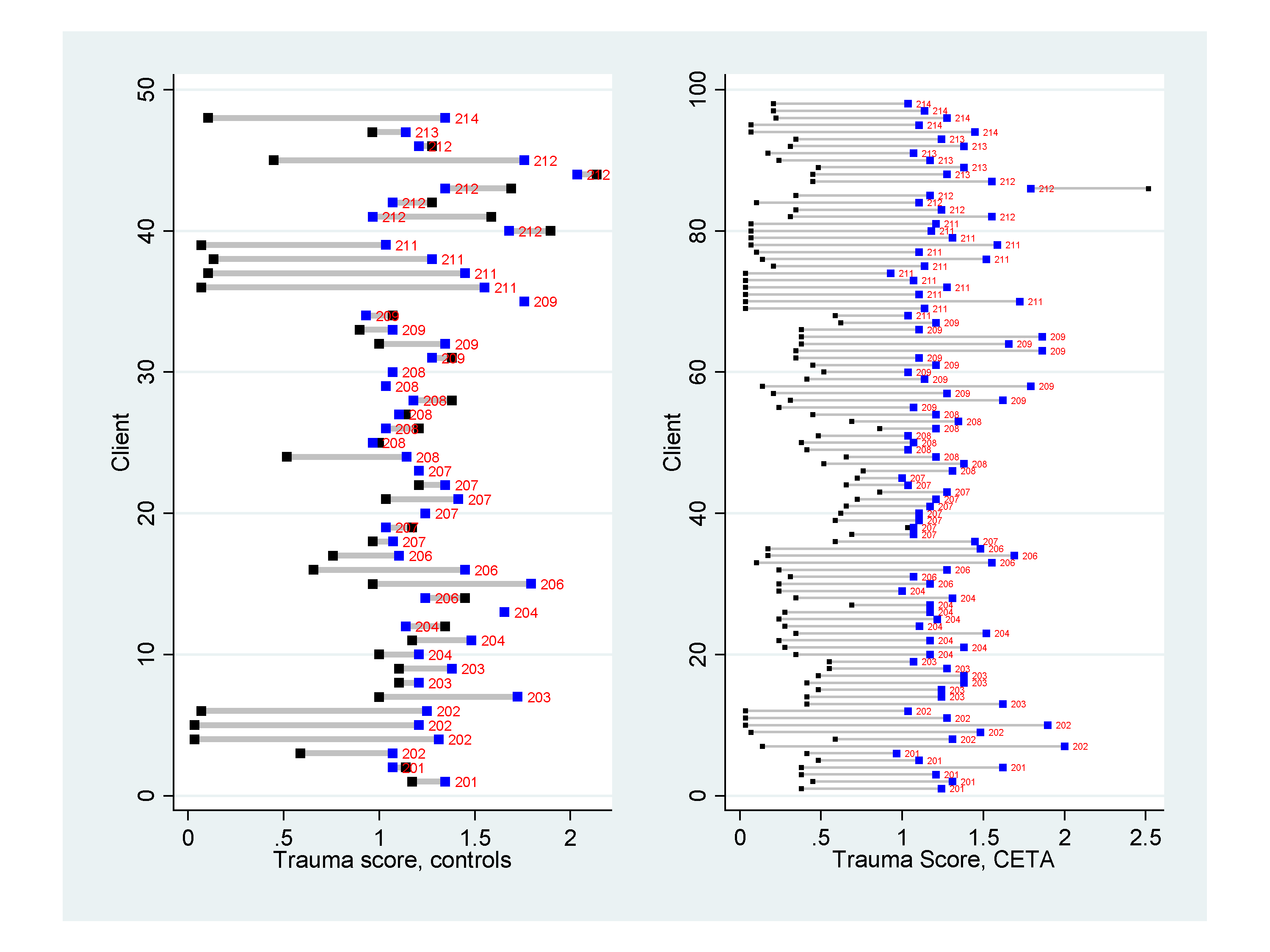

Supplement: Additional file 1: — Figure S1. Baseline and follow up trauma scores for intervention and control clients by CMHW. The plot on the left shows the baseline and final trauma symptom scores of controls and the plot on the right shows the symptom scores of clients who received CETA. Baseline scores are highlighted with blue symbols; follow up scores have black symbols. Grey lines connect the baseline with the follow up score and the length reflects the amount of change. Red text indicates the CMHW who enrolled the control clients and who provided therapy to the CETA clients. The distribution of scores does not suggest any systematic manipulation of scores in a way to create the large effect size found in this trial. Baseline symptom scores vary across clients, and vary within the clients of the CMHWs. Follow up scores also vary across and within the clients of CMHWs, with a few exceptions. Where a particular CMHW has a group of intervention clients with similar follow up scores (e.g., 202, 211), the controls have similar follow up scores to that CMHW’s intervention clients. If there was an effort by these two CMHWs to artificially create a large effect size, we would expect the opposite pattern: intervention clients would have very different and lower follow up scores than controls. Figure S2. Baseline and follow up trauma scores for intervention and control clients by follow up interviewer. The plot on the left shows the baseline and final trauma symptom scores of controls and the plot on the right shows the scores of clients who received CETA. Baseline symptom scores are highlighted with blue symbols; follow up scores have black symbols. Grey lines connect the baseline with the follow up score and show the amount of change between the two time points. Green text indicates the interviewer who conducted the follow up interview for a particular client. As in Figure S1, the distribution of scores does not suggest any systematic manipulation of scores in a way to create the large effect size f [file 12888_2015_622_MOESM1_ESM.zip › 1430994316159765_fig3.tiff]

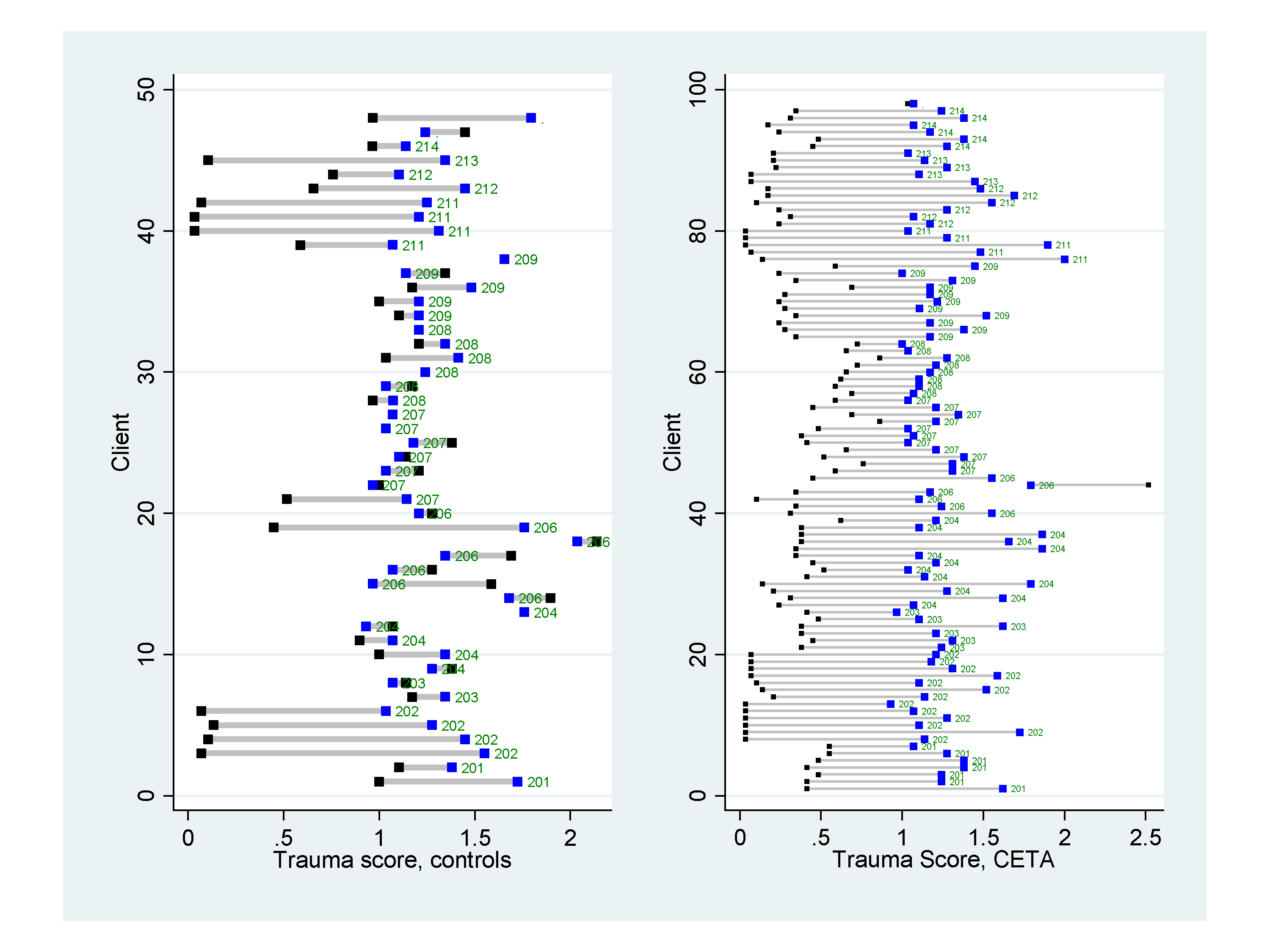

Supplement: Additional file 1: — Figure S1. Baseline and follow up trauma scores for intervention and control clients by CMHW. The plot on the left shows the baseline and final trauma symptom scores of controls and the plot on the right shows the symptom scores of clients who received CETA. Baseline scores are highlighted with blue symbols; follow up scores have black symbols. Grey lines connect the baseline with the follow up score and the length reflects the amount of change. Red text indicates the CMHW who enrolled the control clients and who provided therapy to the CETA clients. The distribution of scores does not suggest any systematic manipulation of scores in a way to create the large effect size found in this trial. Baseline symptom scores vary across clients, and vary within the clients of the CMHWs. Follow up scores also vary across and within the clients of CMHWs, with a few exceptions. Where a particular CMHW has a group of intervention clients with similar follow up scores (e.g., 202, 211), the controls have similar follow up scores to that CMHW’s intervention clients. If there was an effort by these two CMHWs to artificially create a large effect size, we would expect the opposite pattern: intervention clients would have very different and lower follow up scores than controls. Figure S2. Baseline and follow up trauma scores for intervention and control clients by follow up interviewer. The plot on the left shows the baseline and final trauma symptom scores of controls and the plot on the right shows the scores of clients who received CETA. Baseline symptom scores are highlighted with blue symbols; follow up scores have black symbols. Grey lines connect the baseline with the follow up score and show the amount of change between the two time points. Green text indicates the interviewer who conducted the follow up interview for a particular client. As in Figure S1, the distribution of scores does not suggest any systematic manipulation of scores in a way to create the large effect size f [file 12888_2015_622_MOESM1_ESM.zip › 1430994316159765_fig4.tiff]
